# In Vitro Approaches to Explore the Anticancer Potential of One Natural Flavanone and Four Derivatives Loaded in Biopolymeric Nanoparticles for Application in Topical Delivery Treatments

**DOI:** 10.3390/pharmaceutics15061632

**Published:** 2023-05-31

**Authors:** Paola Bustos-Salgado, Berenice Andrade-Carrera, Valeri Domínguez-Villegas, Véronique Noé, Mireia Mallandrich, Helena Colom, Ana Calpena-Campmany, María Luisa Garduño-Ramírez

**Affiliations:** 1 Departament de Farmàcia i Tecnologia Farmacèutica, i Fisicoquímica, Facultat de Farmàcia i Ciències de l’Alimentació, Universitat de Barcelona (UB), Av. Joan XXIII 29-31, 08028 Barcelona, Spain; paola_bustos_salgado@ub.edu (P.B.-S.); bereniceac@uaem.mx (B.A.-C.); anacalpena@ub.edu (A.C.-C.); 2 Centro de Investigaciones Químicas, Instituto de Investigación en Ciencias Básicas y Aplicadas, Universidad Autónoma del Estado de Morelos, Av. Universidad 1001, Cuernavaca 62209, Morelos, Mexico; lgarduno@uaem.mx; 3 Institut de Nanociència i Nanotecnologia, Universitat de Barcelona (UB), 08028 Barcelona, Spain; 4 Facultad de Nutrición, Universidad Autónoma del Estado de Morelos, Calle Iztaccihuatl S/N, Col. Los Volcanes, Cuernavaca 62350, Morelos, Mexico; 5 Facultad de Ciencias Químicas e Ingeniería, Universidad Autónoma del Estado de Morelos, Av. Universidad 1001, Cuernavaca 62209, Morelos, Mexico; valeri.dominguez@uaem.mx; 6 Departament de Bioquímica i Fisiologia, Facultat de Farmàcia i Ciències de l’Alimentació, Universitat de Barcelona (UB), 08028 Barcelona, Spain; vnoe@ub.edu

**Keywords:** *Eysenhardtia platycarpa*, flavanone derivatives, polymeric nanoparticles, cytotoxicity activity, ex vivo permeation, SAR study, in silico anticarcinogenic analysis

## Abstract

The increasing number of skin cancer cases worldwide and the adverse side effects of current treatments have led to the search for new anticancer agents. In this present work, the anticancer potential of the natural flavanone 1, extracted from *Eysenhardtia platycarpa*, and four flavanone derivatives 1a–d obtained by different reactions from 1 was investigated by an in silico study and through cytotoxicity assays in melanoma (M21), cervical cancer (HeLa) cell lines and in a non-tumor cell line (HEK-293). The free compounds and compounds loaded in biopolymeric nanoparticles (PLGA NPs 1, 1a–d) were assayed. A structure–activity study (SAR) was performed to establish the main physicochemical characteristics that most contribute to cytotoxicity. Finally, ex vivo permeation studies were performed to assess the suitability of the flavanones for topical administration. Results revealed that most of the studied flavanones and their respective PLGA NPs inhibited cell growth depending on the concentration; 1b should be highlighted. The descriptors of the energetic factor were those that played a more important role in cellular activity. PLGA NPs demonstrated their ability to penetrate (*Q_p_* of 17.84−118.29 µg) and be retained (*Q_r_* of 0.01−1.44 g/g_skin_/cm^2^) in the skin and to exert their action for longer. The results of the study suggest that flavanones could offer many opportunities as a future anticancer topical adjuvant treatment.

## 1. Introduction

The skin is the largest organ of the human body. External agents can harm it or give rise to some illnesses, such as cancer [1,2]. Skin cancer refers to the abnormal growth of skin cells. It manifests itself mainly in the areas of the skin most exposed to UV radiation, but it is not exclusive. The most prevalent types of skin cancer are basal cell carcinoma and squamous cell carcinoma, originating from keratinocytes, also called non-melanoma skin cancer. They usually grow slowly, and it is not common for them to spread to other parts of the body if they are not treated in time. However, it is melanoma, another type of cancer originating from pigment cells of the skin, which, though it is the least common, can be fatal to humans if it is not treated [3,4,5,6]. The normal treatment in these cases includes the localized excision of the localized disease, chemotherapy and radiation. Today, a great number of studies are concentrating on the search for new treatments or to improve the existing ones, the objective being to reduce or to eliminate the adverse effects, to make the drugs act more specifically and to ensure that they combine better with the current pharmaceutical drugs or treatments. With regards to this, natural products could offer the possibility of obtaining novel molecules and evaluating their anticancer potential as an adjuvant anticancer agent [7]. To screen natural products in terms of their anticancer molecules, researchers use both raw extracts and isolated compounds for in vitro tests. Various studies already published on natural plant derivatives have shown their potential in the treatment of cancer [8,9,10,11,12,13].

Another of the strategies in the development of anticancer candidates consists of the development of formulations. As part of this line of research, we must include the systems of the administration of novel formulations. Included in this approach, we can find the systems for the administration of nanoscale pharmaceutical drugs (liposomes, nanoparticles, solid lipids, nanoemulsions and polymeric nanoparticles (NPs), among others). Nanoparticles offer advantages over other current treatments to alleviate cancer, as they allow the active compounds to reach the site of action in therapeutic concentrations and to remain there longer and so achieve a greater effect [4,14,15,16]. These systems have been described in the related literature, and some results show that the NPs are capable of transporting the molecules through the cutaneous barrier, prolonging their liberation and improving the penetration in comparison to non-encapsulated molecules [5]. Some studies have demonstrated that the magnetic NPs made up of albumin, PLGA and 5-fluoracil exercise greater therapeutic effects than the semi-solid formulation of 5-fluorouracil, diclofenac, imiquimod and the photodynamic therapy, which has been commonly used to treat skin cancer [5,17]. Furthermore, the low solubility of the natural compounds means that their bioavailability is restricted due to their chemical structure. However, this drawback can be overcome using nanostructured formulations [11,18]. The topical administration of drugs through the skin make it possible to realize therapies with localized treatments aimed at allowing in situ action. The NPs can be effective in treatments supporting the aforementioned. The topical administration of NPs to transport the anticancer drugs is an interesting alternative to strengthen the therapeutic benefits, as well as to reduce the toxicity in normal tissue [3].

**Figure 1:**
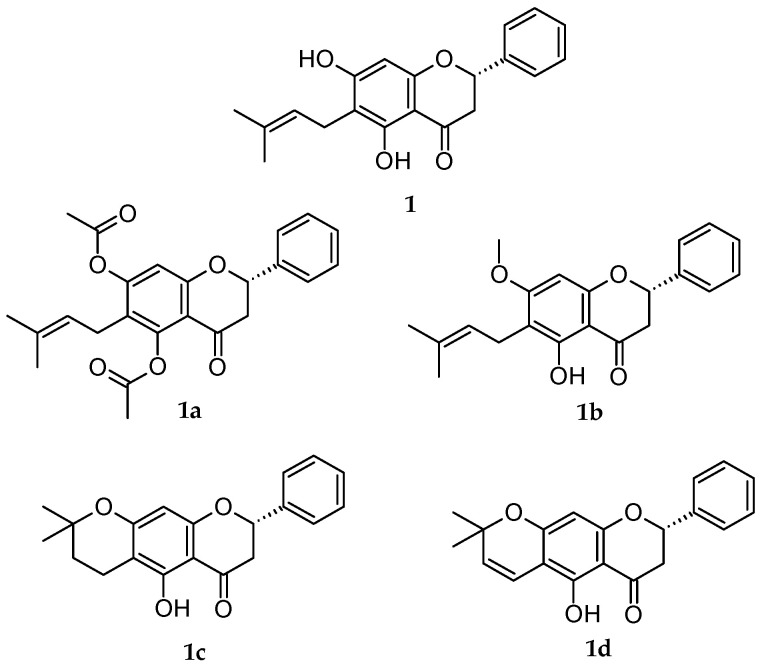
Chemical structures of the analyzed flavanones.

Recently, our research group isolated specific flavanones from extracts of *Eysenhardtia platycarpa* leaves [19], and synthesized derivatives were created through different reactions [20] to probe the antioxidant properties [21], anti-inflammatory [22] and cytotoxicity effects in MiaPaCa pancreatic cancer cells (Figure 1) [19]. *E. platycarpa* is a species that belongs to the *Fabaceae* family, found across all of Mexico; it is commonly called “cuate”, “palo dulce” or “palo azul”. It has been used as a herbal remedy in the treatment of kidney and liver diseases. Phytochemical investigations have revealed its presence in a great variety of secondary metabolites, among others flavonoids, polyphenolic compounds and isomeric structures such as flavanones. These types of compounds possess diverse functionalities and therapeutic attributes [23].

In light of all the foregoing information, the purpose of this investigation was to evaluate the cytotoxic capacity of natural flavanone (1) and four derivatives of 1 (1a, 1b, 1c, 1d) (Figure 1) and their corresponding PLGA NPs. An online analysis was carried out using *PASS Online*, with free access to discover if the flavanones gave results similar to those of the drugs and if they had drug-like characteristics or not before an in vitro analysis. The next step was to try out the free and formulated flavanones on the melanoma cell lines (M21), cervical cancer (HeLa) and non-tumoral embryo human kidney (HEK-293). With the results obtained, a study of the structure/activity relationships was set up (PLGA NPs). This study also explored the capacity of these substances to penetrate human skin and thus find a clinical application to fight skin cancer.

## 2. Materials and Methods

### 2.1. Chemicals

The analytical grade solvents (ethanol, methanol and acetonitrile) and reagents for all the procedures described in this paper were purchased from Sigma-Aldrich (Madrid, Spain) and Thermo Fisher Scientific (Barcelona, Spain). MilliQ^®^ Plus System lab (Millipore Corporation, Burlington, MA, USA) supplied the distilled water used in the experiments.

### 2.2. In Silico Analyses

To carry out a theoretical evaluation of the anticarcinogenic properties of the flavanones, the Molinspiration^®^ server (http://www.molinspiration.com, accessed on 18 September 2022) and *PASS Online* (Prediction of Activity Spectra for substance, http://way2drug.com/PassOnline, accessed on 18 September 2022) were used. They contain 4099 different biological activities and were used for a theoretical evaluation of the anticancarcinogenic properties of the flavanones in the study. *PASS Online* permits the determination of the activity specter of the compounds in terms of probable activity (*Pa*) on a scale of results from 0 to 1 [24,25]. To use the web platforms, it must be assured that the scheme correctly draws the chemical structures, taking into consideration the spatial set out of each substitute and function present and considering the configuration of the stereogenic (*R*) and (*S*). Therefore, the Simplified Molecular Input Line Entry System (SMILES) was used to guarantee the interpretation in 3D of the organic molecule to be analyzed. This is increasingly being used across the world in the bioactivity prediction of new molecules with pharmacological potential [26].

### 2.3. Poly DL-Lactide-Co-Glycolide Acid (PLGA) Nanoparticles (NPs)

Poly DL-lactide-co-glycolide acid (PLGA) nanoparticles (NPs) of natural flavanone 1 and its derivatives 1a–1d (NP 1, NP 1a- NP 1d) were transferred by Andrade-Carrera’s team et al. [20]. As detailed in their study, firstly, the natural flavanone was obtained (2*S*)-5,7-Dihydroxy-6-(3-methyl-2-buten-1-yl)-2-phenyl-2,3-dihydro-4*H*-1-benzopyran-4-one (1) (CAS No.: 55051-77-9) (Figure 1) [27]. In sum, the leaves of *E. platycarpa* were collected, dried and pulverized. Subsequently, a methanolic extraction (100 g of dried vegetable material per 1000 mL of methanol) was carried out. Compound 1 was isolated from the methanolic extract by reduced pressure chromatography using silica gel. Then, it was purified and characterized by thin-layer chromatography. Secondly, the flavanone derivatives (2*S*)-5,7-bis(acetyloxy)-6-(3-methyl-2-buten-1-yl)-2-phenyl-2,3-dihydro-4*H*-1- Benzopyran-4-one) (1a); (2*S*)-5-hydroxy-7-methoxy-6-(3-methyl-2-buten-1-yl)-2-phenyl-2,3-dihydro-4*H*-1-Benzopyran-4-one) (1b); (8*S*)-5-hydroxy2,2-dimethyl-8-phenyl-3,4,7,8-tetrahydro-2*H*,6*H*-Benzo [1,2-*b*:5,4-*b* 0] dipyran-6-one) (1c); and (8*S*)-5-hydroxy-2,2-dimethyl-8-phenyl-7,8-dihydro2*H*,6*H*-Benzo [1,2-*b*:5,4-*b* 0] dipyran-6-one) (1d) (Figure 1) were obtained, respectively, from natural flavanone 1 through esterification, methylation, cyclization and vinylogous-cyclization reactions (yield reactions were: 1a: 79.6 %, 1b: 58 %, 1c: 87.2%, 1d: 52%) [20]. Finally, to obtain the PLGA NPs for each flavanone studied, 1, 1a, 1b, 1c and 1d, the solvent displacement technique was followed [20]. Accordingly, a 50:50 organic solution of poly (DL-lactide-co-glycolide acid) (PLGA, 90 mg) in acetone (25 mL) containing the respective flavanone (1.0 mg/mL) was gently stirred into an aqueous solution of Poloxamer 188 (P188). Subsequently, acetone was evaporated, and the volume of the NPs was reduced using a B-480 rotary evaporator (Büchi, Labortechnik AG, Flawil, Switzerland). The resultant NPs were frozen and sterilized for in vitro study. Andrade-Carrera and collaborators [20] conducted the physicochemical characterization of the flavanones’ PLGA NPs by means of particle size (Z-average, nm), polydispersity index (PI), zeta potential (Z, mV) and the percentage of entrapment efficiency (EE %). The values of each parameter are listed below (Table 1):

### 2.4. Chromatographic Operating Conditions

The high-performance liquid chromatography (HPLC) analysis was carried out using a previously validated system [28] composed of a Waters 515 HPLC pump with a 717 Plus autosampler, a dual λ absorbance UV-vis 2487 detector (Waters, Milford, MA, USA) and an analytical column Atlantis^®^ C18 5 μm 250 mm × 4.6 mm. The chromatographic separation was achieved using the isocratic elution method with 10 μL of sample injection volume at room temperature. The mobile phase consisted of W-water and AcN-acetonitrile (% W: % AcN) at different proportions depending on the flavanone (1 (30:70), 1a (20:80), 1b (40:60), 1c (20:80), 1d (10:90)), with a flow rate of 1 mL/min. The detection wavelengths were 300 nm for almost all flavanones except for 1a with 320 nm. The peak area was used to quantify each analyte.

### 2.5. Cytotoxicity Assays: Cell Culture and Cell Viability Assays

Human embryonic kidney (HEK-293), melanoma (M21) and cervical cancer (HeLa) cell lines were obtained from the cell bank resources of the University of Barcelona. Cell lines were routinely grown in F12 medium (Gibco, Grand Island, NY, USA), supplemented with 10% (*v*/*v*) fetal bovine serum (Gibco), 100 U/mL sodium penicillin G and 100 µg/mL streptomycin and controlled at 37 °C in a 5% CO_2_ humidified atmosphere. Flavanones and NP flavanones were dissolved in dimethyl sulfoxide (DMSO) before being added to cell incubations, and the final concentration of DMSO in the culture medium was always lower than 1% (*v*/*v*). On the day of the experiments, cells were placed in 96-well dishes with 200 µL of F12 medium and, 2 h later, incubated with flavanones (1, 1a–d) at 5, 10, 25 and 50 µM, and NP 1 and NP 1a–d at 10, 50, 75 and 100 µM. Cell viability was determined by the MTT assay five days later. For this, 20 µL of a 0.5 mg/mL solution of MTT [3-(4,5-dimethylthiazolyl-2)-2,5-diphenyltetrazolium bromide] in PBS and 20 µL of a 50 mM succinic acid solution in PBS (Sigma-Aldrich, Barcelona, Spain) were added to the culture dishes and incubated for 3 h at 37 °C. Then, the dark blue crystals were dissolved in a 10 % sodium dodecyl sulfate (SDS) solution in DMSO. Finally, the absorbance was measured at 570 nm in a Modulus Microplate spectrophotometer (Turner BioSystems, Madrid, Spain). The control condition corresponded to the absorbance readings from cells without any treatment, and the Control + DMSO indicated the effect of DMSO by itself on cell viability. Cell viability results were expressed as the percentage of cell survival with respect to the control cells grown in the absence of both flavanones and NPs flavanones (Equation (1)).

(1) $$\mathrm{Cell}\;\mathrm{viability}\;\left(\%\right)=\frac{\mathrm{Sample}\;\mathrm{absorbance}}{\mathrm{Control}\;\mathrm{absorbance}}\times 100$$

IC_50_ values were calculated using the GraphPad Prism 5 software (GraphPad Software, Inc., San Diego, CA, USA).

### 2.6. Structure–Activity Relationship Study (SAR)

For the calculation of some physicochemical parameters, such as molecular geometry; the heat of formation; the highest occupied molecular orbital (HOMO); and the lowest unoccupied molecular orbital (LUMO) energy, volume, mass, dipole moment and area, the AM1 semi-empirical method was used, taken from the Hyper Chem 8.0 program package. The analysis of cytotoxicity activity in vitro results and their correlation with physicochemical parameters was obtained using the multivariable linear regression analyses with Sigma Stat Software (SPSS 26.0, Chicago, IL, USA).

### 2.7. Ex Vivo Studies

Permeation experiments were performed to assess the amount of each flavanone from NP formulation that can cross human skin and can be retained within the tissue. To achieve this, the Franz-type diffusion cells (FDC 400, Crown Glass, Somerville, NY, USA) were used. Three determinations were performed in parallel. Human skin (400 μm thickness) from the abdominal region of healthy plastic surgery patients was used as a permeation membrane. All the skin samples used in the study were tested to demonstrate the integrity of the barrier function of the stratum corneum by measuring the trans-epidermal water loss (TEWL−Dermalab) [29], and this resulted in normal values of around 20 g/h/m^2^. The receptor chamber was filled with ethanol: water (70:30) solution. The cells were kept at 32 °C using a temperature-controlled circulating bath. The dose applied in the donor compartment was 300 µL of the respective flavanone NPs in a diffusion area of 2.54 cm^2^ (*n* = 3 for each NP). Aliquots of 300 µL were collected from the receptor compartment at different times for 24 h, and the same volume of ethanol: water (70:30) solution was added to the receptor chamber. The number of flavanones permeated (*Q_p_*) through human skin was determined by HPLC analysis described in the section Chromatographic Operating Conditions.

At the end of the experiment, the amount of flavanone retained in the skin membranes (skin retention, *Q_r_*, μg/g skin/cm^2^) was determined. Firstly, it was necessary to quantify the amount of flavanone that could be extracted *Q_ext_* (μg/g) in accordance with the extraction method of the skin study. To achieve this, the skin was removed from the Franz cells and rinsed with 0.05% solution of dodecylsulphate and distilled water in the final stage. The permeation areas of the assayed skin were excised, perforated with mechanical puncture to facilitate the exit of the flavanone from the tissue, and weighed. Then, the flavanone contained therein was extracted with ethanol: water (70:30) mixture in an ultrasonic processor for 20 min. Finally, the solutions were measured by HPLC to obtain the *Q_ext_* data as described before. To finish, the *Q_r_* value was estimated considering the recovery percentage (R%) calculated in previous studies [27] following Equation (2):

(2) $$Q_{r}=\frac{Q_{ext}}{R\%}*100\;$$

### 2.8. Data Analyses

Statistical analyses were performed using the statistical package GraphPad Prism 5 (GraphPad Software, Inc., San Diego, CA, USA) for in vitro and ex vivo studies (Section 2.5 and Section 2.7). Data significance was evaluated by applying the one-way analysis of variance (ANOVA) with the Bonferroni post hoc test, and *p* < 0.05 was considered statistically significant. For the SAR studies, the spreadsheet program Microsoft Excel 2010 was used to obtain a multiple linear regression analysis and the value of the F statistic (for Section 2.6). All assays were carried out in triplicate, and the data were presented as mean ± SD.

## 3. Results

### 3.1. In Silico Analyses

The in silico studies made it possible to predict and evaluate flavanones 1, 1a–d before performing in vitro tests. The probability value of a compound being active (*Pa*) as an anticarcinogenic or antineoplastic agent using *Pass Online* program for all flavanones (1, 1a–d) is shown by the results to be above or around 0.7 (Table 2). In the case of some proteins affected in cancer cases, such as matrix metalloproteinase-9 (MMP-9) and Caspase-3, only flavanones 1a and 1b exhibited a *Pa* close to 0.7. For other important cancer indicators, such as Caspase-8, Telomerase, Interleukine-6 and Interleukine-10, the *Pa* of all flavanones was lower.

### 3.2. Cytotoxicity Activity

**Figure 2:**
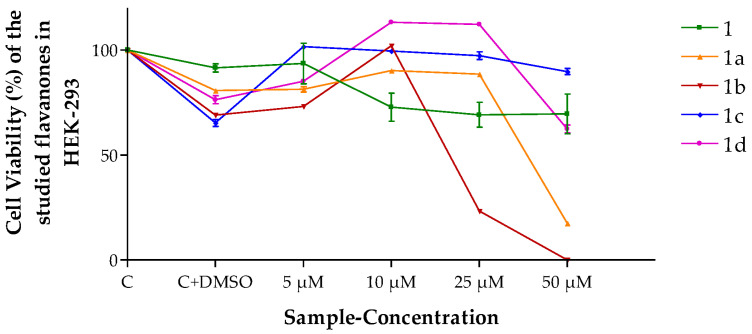
Cell viability (%) of the different flavanones in the HEK-293 cell line. Mean ± SD (*n* = 3). C = Control (cells without any treatment). C + DMSO = cells incubated only with DMSO.

Cancer cell lines are usually the first option to evaluate the anticancer potential of new molecules; among these, HeLa is the most used. Additionally, the HEK-293 cell line was used in the in vitro assays with the purpose of evaluating the effect of flavanones on a non-tumor human cell line. Results in Figure 2 show the effect of flavanones on HEK-293 cell viability dependent on their concentration in the culture medium. Flavanone 1b yielded the highest cytotoxicity at 50 μm.

**Figure 3:**
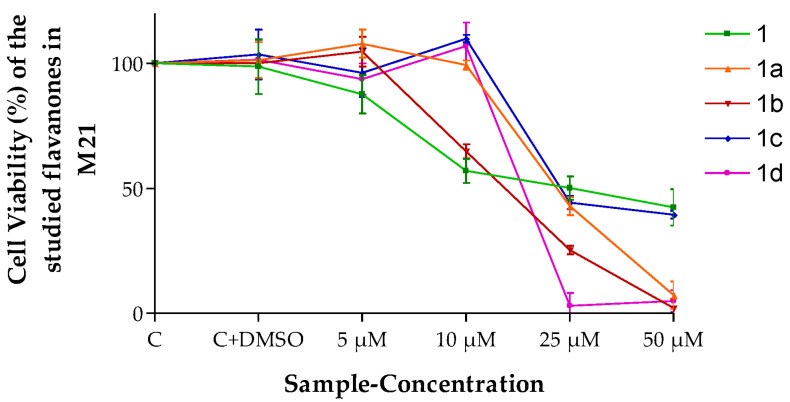
Cell viability (%) of the different flavanones in the M21 cell line. Mean ± SD (*n* = 3). C = Control (cells without any treatment). C + DMSO = cells incubated only with DMSO.

The results shown in Figure 3 indicate that flavanones 1b and 1d caused a lower decrease in cell viability than 1, 1a and 1c at the highest concentration tested (50 µM) when they were evaluated in melanoma M21 cells.

**Figure 4:**
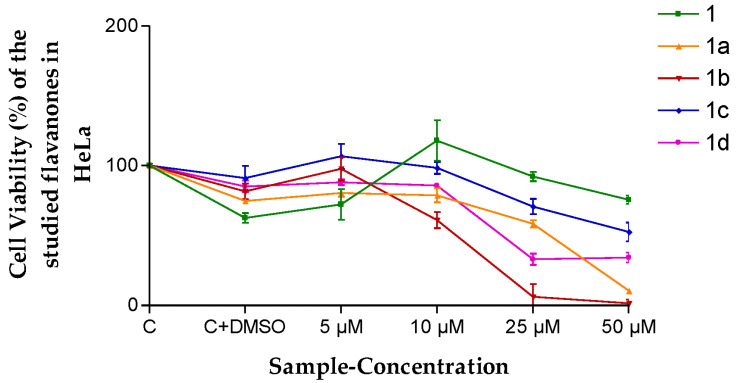
Cell viability (%) of the different flavanones in the HeLa cell line. Mean ± SD (*n* = 3). C = Control (cells without any treatment). C + DMSO = cells incubated only with DMSO.

In the case of HeLa cells, once more, flavanone 1b presented the highest cytotoxicity in the range of 10 to 50 µM (Figure 4).

**Figure 5:**
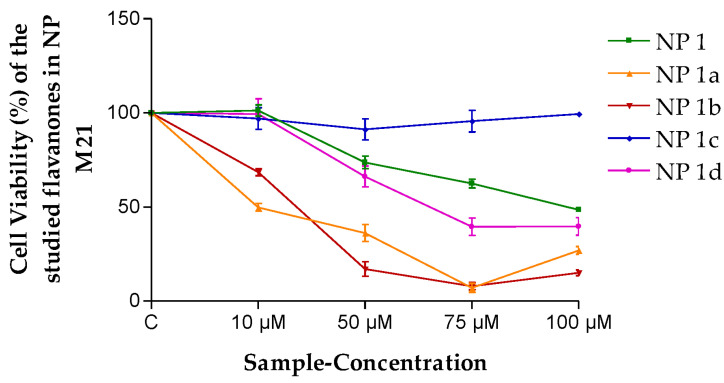
Cell viability (%) of the different flavanones NPs in the M21 cell line. Mean ± SD (*n* = 3). C = Control (cells without any treatment).

On the other hand, the PLGA NP formulation without any flavanone (NP Blank) was evaluated, and no toxicity was observed. Therefore, the flavanone NPs were tested in M21 and HeLa cell lines, as were the flavanones solutions. As can be seen in Figure 5, NP 1 presented a moderated effect on cell viability (48.54%) at a concentration of 100 µM in M21 cells. For NP 1a and NP 1b, a dose-dependent decrease in cell viability was observed up to 75 µM.

**Figure 6:**
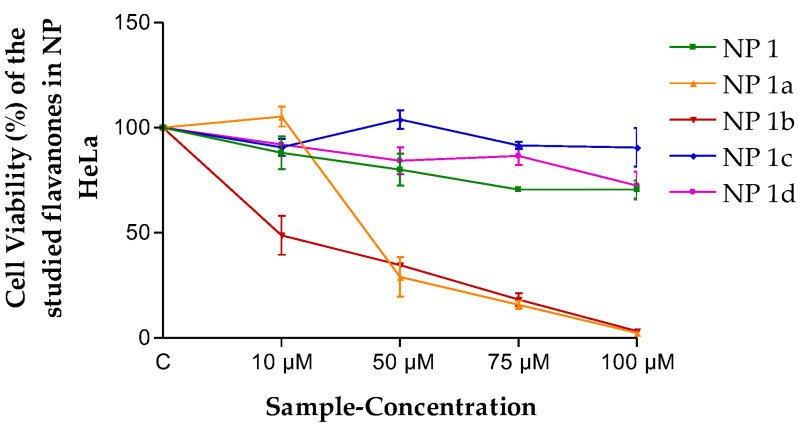
Cell viability (%) of the different flavanone NPs in the HeLa cell line. Mean ± SD (*n* = 3). C = Control (cells without any treatment).

In the viability assays in HeLa cells with flavanones NPs (Figure 6), NP 1a and NP 1b were more cytotoxic than all the other formulations, and no significant cell growth inhibition was observed for NP 1, NP 1c and NP 1d.

The IC_50_ values were calculated for all flavanones (1, 1a–d) and PLGA NPs (1, 1a–d) in HEK-293, M21 and HeLa cells (Table 3). A high value reflects a low cytotoxicity effect. No significant effect in cell viability for NP 1c was observed at the different concentrations assayed. Therefore, no IC_50_ was calculated in this case (NE).

### 3.3. Structure–Activity Relationship Study (SAR)

To establish which of the most relevant flavanone physicochemical properties contributed to the observed cytotoxicity activity, it was necessary to correlate the flavanone physicochemical properties and the pharmacological activity obtained in vitro by means of multiple linear regression. The correlation coefficient r^2^ and the value of the F statistic were considered in terms of whether or not they demonstrated a significant correlation. Firstly, some physicochemical properties of flavanones were calculated through their 3D structure, as drawn in the HyperChem program (Table 4).

Considering that data closer to 1 in r^2^ indicates a better correlation and that the greater the value of the Fisher F statistic, the more the variation in the correlation of the descriptors versus the results of cell viability, the more it indicates the possible cause of their cytotoxicity effect. From the results listed in Table 5, it can be observed that the energy descriptor factors (ET, EE, EF) played a more important role in the behavior of the cellular activity, and this behavior is maintained when the flavanones are formulated in NPs, as shown by the results with M21 cells. The HOMO and LUMO (electron transfer) orbital descriptors are the least involved in the cytotoxicity effect of flavanones (very low r^2^ and F values).

In the same way, in HeLa cells, the energy descriptors (ET, EE, EF) are the most important factors in the influence on the flavanones’ biological response. However, when the flavanones were formulated in the NPs, all the descriptors improved their correlation with the cytotoxicity results (r^2^ and F are higher). However, the lipophilicity/hydrophilicity ratio factor (Log *P*, α, μ) stood out as the most relevant factor in affecting the biological activity tested.

### 3.4. Ex Vivo Studies

The flavanone NPs exhibited a different behavior when they were assayed in human skin in an ex vivo study. NP 1a presented a much higher flux value than the rest of the NPs (160.98 µg/h/cm^2^, Table 6,), while the lowest flux was developed by NP 1b. In the same way, NP 1a gave the highest retention in human skin in Franz cells. Nevertheless, NP 1d was the formulation that allowed the largest amount of flavanone to permeate (118.29 µg).

## 4. Discussion

In silico studies are valued tools in pharmaceutical research, as they enable scientists to hypothesize about molecules for a particular disease [31]. Furthermore, in silico parameters have a significant role in the estimation of the biological activity in the human body [32,33]. The prediction of Activity Spectra for Substances (*PASS Online*), one of the many different server webs that exist, allows the sifting of chemical compounds through databases and, therefore, avoids dedicating an unnecessary effort to the inactive molecules. In their in silico studies, Ahmed Hasan Abkar et al. discovered that the bioactive compounds β-asarone, methyl-piperonylketone and coumaric acid obtained from *Piper crocatum* act against cancer through the inhibition of the tumor necrosis factor alpha protein (TNF-α) and the Matrix metallopeptidase protein (MMP9) [34]. In the current study, we found that all flavanones evaluated showed a probability greater than 0.7 of being active (*Pa*) as anticarcinogenic and antineoplastic agents (Table 2). These *Pa* values show that the chances of finding experimental activity are rather high [24]. The flavanone derivatives 1a, 1b and 1d exhibited higher *Pa* values than the natural flavanone 1. Some results have evidenced that flavonoids could stimulate cell death pathways through the targeting of the apoptotic signaling cascade by the activation of some proteins, such as Caspase -3, -6, -8 and -9 [35,36]. Furthermore, it is known that the MMP-9 expression is implicated in apoptosis, invasion and metastasis [37]. With regards to this, of all the flavanones evaluated, 1a and 1b showed higher probabilities for the MMP-9 expression inhibitor. In the same way, 1a and 1b showed the best *Pa* to activate Caspase-3. These in silico flavanone predicted outcomes were endorsed by the in vitro cytotoxicity studies. It is common to use cancer cell lines as the first study to evaluate molecules that may have a possible antineoplastic activity [13]. Our assays are intended to evaluate the activity of the flavanones against various cell lines as a preliminary study to focus on their topical application in anticancer treatments. The results in our work indicate that the highest cytotoxicity activity among the tested compounds was exhibited by flavanone 1b. Furthermore, the most sensitive cell lines were observed with the NP 1b treatment. In our group’s previous studies, these flavanones were evaluated against the MiaPaCa-2 cell line, and the results showed that flavanones 1a and 1d were the best in decreasing cell viability [20]. These results are in agreement with the literature since Lei Chen et al., reported evidence that sustains that the *O*-methylation of flavonoids makes them metabolically more stable and, therefore, increases their bioavailability as well as the growth of tissue distribution compared with unmethylated forms [38]. Additionally, some chalcones and flavanones with a methoxyl moiety in the A ring presented activity against the A549 (human lung carcinoma) cell line; whereas the same compounds without methoxyl groups did not [39]. Eman Assirey et al., in the human colon carcinoma (HCT)-116 cell line, showed better inhibitory effects with a methoxy or hydroxy substituent at the C-7 position of the flavanone [40]. This is precisely the case with the flavanones of the study, in which 1b possesses a methoxyl substituent at C-7. On the other hand, some studies reported that the hydroxylation in 5- and 7 of the A ring of flavonoids were shown to be beneficial in their antioxidant activity [38]. In addition, the capacities of flavonoids as antioxidants contribute to their being candidates for the prevention of cancer growth [40]. It is worth noting that flavanone 1b has a hydroxyl-moiety in C-5 (Figure 1). The cytotoxicity assays carried out with the free flavanones are indicative as a preliminary result since it would not be possible to use the compounds directly as a treatment in some tissues, such as skin or mucosa or in the vagina, since they are dissolved in DMSO. The studies, which indicate the real efficacy of the treatment, are those in which the formulations do contain flavanones in NPs (as these can be administered directly on the tissue).

Structure–activity relationship studies (SAR) sought to correlate molecular structures with their properties and biochemical activities. The molecular properties calculated for the activity correlation are easily obtained with the computational chemistry package HyperChem [41]. It is said that the compound with the minimum binding energy will have the maximum binding affinity. Therefore, that compound would be the best candidate for developing a drug [32]. All the flavanone derivatives (1a–d) possess more negative binding energy than the natural flavanone 1. According to the most negative value, we could consider a priori flavanone 1a with −5879.46 kcal/mol as the most effective. The surface area is also an important parameter when we want to predict biological activity. The possibility of killing more pathogens grows the greater the charge surface area of a molecule. Further, the charged distribution from the electrostatic potential is related to the surface area [32]. Therefore, higher biological activity is deemed as such when a greater positive charge surface is presented. As seen in Table 4, flavanone 1a and flavanone 1b have bigger surfaces than the natural flavanone 1 and the other derivatives (1c and 1d). The Log *P* (octanol/water partition coefficient) plays an important role in biochemical interactions and bioactivity [42]. The optimal values for the partition coefficient are neither too hydrophobic (lipophilic; the positive value of Log *P*) nor too hydrophilic (lipophobic; the negative value of Log *P*) [32]. From the results obtained, some conclusions can be drawn regarding structure–activity relationships: the energy parameters for free flavanones have an influence on the inhibition of cell growth. On the other hand, the lipophilicity/hydrophilicity ratio factor was very relevant when those flavanones were formulated into NPs. With the results obtained in this work, we can infer that the structural modifications made to the natural flavanone 1 offer a path forward for the development of new molecules with possible anticancer potential.

In order to overcome the low solubility in water, the poor absorption and the bioavailability problems of flavonoids, the advances in nanotechnology delivery systems offer an opportunity in which the use of nanoparticle carriers will be beneficial [43]. Nanoparticles encapsulate molecules into vesicles with nano-size acting as a protector against degradation and as a functionality enhancer. However, the storage stability and slow-release effect can also be improved [44,45]. Therefore, NPs can prolong the time of the action by the drug carried, enhance drug efficacy and reduce adverse reactions [46]. Diverse studies showed the effectiveness of liposomes; poly-ethylene glycol (PEG) liposomes; and nickel-based, lecithin-based and nanoribbon of quercetin in terms of drug delivery into solid tumors of in vitro and in vivo models of varied cancers [36]. Maity et al. mentioned the enhancer effects of catechin and quercetin-based nanoparticles in cancer treatments [47]. The synthetic PLGA has also been widely used to develop NPs in biomedical applications as carriers of active ingredients, which treat different health conditions. Their power as biodegradable substances and the ease of their distribution make them non-toxic and safe for humans [48,49]. PLGA NPs have the structure of a hydrophilic shell and hydrophobic core. Studies reported that the quercetin PLGA NPs improved their solubility and stability [46]. Furthermore, they are capable of sustaining the flavonoid in blood circulation for longer, reducing toxicity to healthy tissues and improving antitumor efficiency in cancers of the liver, ovary and lung [47]. The flavone apigenin has been used to reduce tumors in the skin, though the apigenin NPs produced better results, which might be attributed to the NPs system itself as it may influence the skin distribution of the formulation after topical application [3]. In our case, the NPs containing the flavanones were prepared with the objective of verifying their anticancer action. For this reason, studies have been carried out focused on the skin. We wanted to demonstrate the ability of NPs to penetrate this tissue. The *Q_r_* values show that the stratum corneum acts as a helpful reservoir in topical treatment as the local depot effect potentiates the duration of the treatment. After the topical application of the NPs, part of the flavanones remain in the epidermis and dermis to provide a higher concentration of flavanones in the skin, and consequently, an easier target for an effective anticancer treatment. From the experiments to test whether NP flavanones 1a–d can permeate across human skin and be retained within the tissue, it can be concluded that all the flavanones are able to permeate across the skin, and the systemic effects will be negligible if the volume of body water is considered given the *Q_p_*. Therefore, the indications are that they are not going to be compounds with high toxicity. In addition, all flavanones were retained within the skin, being able to exert a local therapeutic action as well. The flavanone amount retained in the skin was significantly higher in the case of NP 1a than for the other NPs (Table 6). In this way, these results suggest that PLGA NPs could be suitable for the encapsulation of flavanones 1a–d, and they will release them in skin environments. Additionally, these findings could also be useful in the design of encapsulated flavanones with cytotoxicity potential for topical local treatment. Therefore, the use of NPs concomitantly with traditional treatments may optimize the outcomes of the treatments.

The results obtained (Figure 3 and Figure 4) lead one to think that the flavanones are intrinsically effective against the cancerous cellular lines evaluated in this study. This means they are optimal for being considered for future antineoplastic treatment. The fact that perhaps this effectiveness was not so high is compensated by the high retention power in the tissue (*Q_r_*, Table 6), exercising a great reservoir effect as they stay in the skin. Therefore, most of them in the final analysis are formidable compounds in terms of better meeting the need evaluated.

In sum, the advantages we find in this manuscript are various. On one hand, the in-silico studies take us to the starting point of the previous in vitro studies. In addition, the modification of the molecular structure of the natural flavanone so as to obtain its derivatives allowed us to corroborate the importance of modifying the leader compounds to come up with a molecular structure more adequate for the cytotoxic action. Moreover, the study carried out in the skin was vital in discovering that this type of compound formulated in PLGA NPs has the capacity to cross skin and to allow the pharmacological action. All this information will be of immense use in future in vivo studies.

## 5. Conclusions

One natural flavanone isolated from *E. platycarpa* and four flavanone derivatives free and loaded in PLGA NPs were screened for their cytotoxicity activity; firstly, in HEK-293 cells to ensure that they would not affect healthy cells or tissues and then, in M21 and HeLa cell lines to test the anticancer potential. The previous in silico screenings were useful to evaluate the flavanones before their in vitro evaluation in cell culture, and the two results were concordant. The cytotoxicity effects provoked by flavanones depended on their structural differences. Total flavanones presented cytotoxicity activity, and their effect was maintained when they were carried by PLGA NPs. Of all the flavanones and formulations, flavanone 1b, NP 1a and NP 1b had the best parameters of cytotoxicity. The skin permeation of the flavanone PLGA NPs was studied to demonstrate their possible local action after local treatment. These studies indicate that the developed nanostructured system is the optimal vehicle for the topical delivery of flavanones, and those NPs have a promising future as potential adjuvant anticancer agents, making a contribution. Taking into consideration that NPs are good precursors of new classic formulations like gels and creams to be administrated by different routes, they could thus form part of preclinical studies. Further studies are required to go beyond the limits of this paper and to elucidate the mechanism of the effect of flavanones.

## Figures and Tables

**Table 1:** Physicochemical characterization of flavanones PLGA NPs 1, 1a–d.

Nanoparticle	Physicochemical Characteristics			
	Z-Average (nm)	PI	Z (mV)	EE (%)
NP 1	205.20 ± 0.27	0.06 ± 0.05	−8.25 ± 0.24	80.00 ± 4.75
NP 1a	178.03 ± 1.33	0.08 ± 0.004	−9.05 ± 0.32	88.47 ± 4.18
NP 1b	141.63 ± 0.78	0.09 ± 0.02	−10.63 ± 0.23	85.00 ± 5.80
NP 1c	175.17 ± 0.60	0.10 ± 0.03	−6.48 ± 0.38	78.28 ± 5.85
NP 1d	173.40 ± 1.59	0.06 ± 0.01	−6.65 ± 0.41	78.75 ± 4.34

Z-average: average diameter of NPs; PI: Polydispersity Index; Z: Zeta potential; EE: entrapment efficiency.

**Table 2:** In silico predicted anticarcinogenic activity score data for chemical structure flavanones **1**, **1a**–**d**.

Data	Flavanone				
	1	1a	1b	1c	1d
Anticarcinogenic (*Pa*)	0.790	0.768	0.797	0.722	0.625
Antineoplastic (*Pa*)	0.774	0.804	0.758	0.692	0.797
MMP-9	0.734	0.588	0.766	0.481	0.523
Caspase-3	0.423	0.648	0.604	0.318	0.330
Caspase-8	0.256	0.297	0.279	ND	0.259
Telomerase	0.136	ND	0.116	0.083	0.121
Inteleukine-6	0.192	0.171	0.179	ND	0.164
Interleukine-10	ND	ND	0.095	0.096	0.099

ND: Not detectable; *Pa*: probability of being active; MMP-9: matrix metalloproteinase-9.

**Table 3:** IC_50_ (µM) value of flavanones and PLGA NPs.

Compound	Cell Line			NPs	Cell Line	
	HEK-293	M21	HeLa		M21	HeLa
	IC_50_ (µM)				IC_50_ (µM)	
1	6.59	7.37	24.05	NP 1	49.72	11.19
1a	28.1	20.88	19.53	NP 1a	10.28	36.73
1b	22.10	13.29	11.14	NP 1b	14.28	10.89
1c	27.21	21.18	19.09	NP 1c	NE	NE
1d	28.28	15.77	12.81	NP 1d	49.71	24.84

NE = no effect observed on cell viability.

**Table 4:** Flavanones physicochemical properties using HyperChem 8.0 program.

Physicochemical Properties	Flavanones				
	1	1a	1b	1c	1d
Total energy (kcal/mol)	−94,946.90	−12,2792.00	−98,527.20	−94,951.30	−94,294.20
Binding energy (kcal/mol)	−4809.71	−5879.46	−5078.27	−4814.19	−4682.68
Heat of formation (kcal/mol)	−111.63	−170.30	−105.10	−116.11	−88.81
Surface area (A^2^)	577.35	680.43	607.10	538.39	549.13
Volume (A^3^)	959.56	1176.83	1014.63	913.85	917.78
Mass (amu)	324.48	408.40	338.40	324.38	322.36
HOMO (eV)	−9.02	−9.11	−8.95	−8.98	−8.67
LUMO (eV)	−0.56	−0.65	−0.50	−0.40	−0.54
Log *P*	0.70	0.26	0.73	0.05	−0.07
Dipole moment (µ)	3.55	1.50	3.82	1.44	1.52
Polarizability (α)	35.81	43.32	37.64	35.22	35.03

amu = atomic mass units; HOMO = the highest occupied molecular orbital; LUMO = the lowest unoccupied molecular orbital. Log *P* = octanol/water partition coefficient.

**Table 5:** Multiple correlation from intrinsic flavanones and NP flavanones: r^2^ values and Fisher F statistic.

Molecular Descriptors	Free Flavanones				NPs Flavanones			
	M21		HeLa		M21		HeLa	
	r^2^	F	r^2^	F	r^2^	F	r^2^	F
ET, EE, EF	0.99	10.98	0.94	2.57	0.99	13.09	0.97	6.07
HOMO, LUMO	0.26	0.07	0.18	0.03	0.39	0.18	0.62	0.64
A, V, MM	0.58	0.17	0.85	0.84	0.94	2.52	0.99	12.71
Log *P*, α, μ	0.46	0.28	0.89	1.24	0.98	6.77	0.99	36.75

ET = Total energy; EE = Binding energy; EF = Heat of formation; A = Area; V = Volume; MM = Molecular mass; Log *P* = Octanol/water partition coefficient; µ = Dipole moment; α = Polarizability.

**Table 6:** Median (maximum–minimum) values of permeation parameters.

	NP 1 *	NP 1a	NP 1b	NP 1c	NP 1d
*J*/sur (μg/h/cm^2^)					
	0.35	160.98	0.03	0.54	2.37
	(0.53−0.005)	(183.52–138.39)	(0.038–0.022)	(0.61–0.49)	(2.71–2.02)
*Q_r_* (g/g_skin_/cm^2^)					
	0.54	1.44	0.07	0.04	0.01
	(0.62–0.46)	(1.66–1.22)	(0.08–0.06)	(0.05–0.03)	(0.006–0.004)
*Q_p_* (µg)					
	30.34	78.08	17.84	43.32	118.29
	(34.13–26.55)	(90.81–65.35)	(20.29–15.40)	(50.12–36.52)	(132.49–104.10)

*: Data obtained from Domínguez et al. [30]; *J*: Flux; *Q_r_*: retained amount at 24 h of flavanones 1 and 1a–d in human skin; sur: surface; *Q_p_*: permeated amount at 24 h of flavanones 1, 1a–d from NPs in human skin.

## Data Availability

The data presented in this study are available on request from the corresponding author.
